# Hepatitis B Virus HBx Activates Notch Signaling via Delta-Like 4/Notch1 in Hepatocellular Carcinoma

**DOI:** 10.1371/journal.pone.0146696

**Published:** 2016-01-14

**Authors:** Pornrat Kongkavitoon, Pisit Tangkijvanich, Nattiya Hirankarn, Tanapat Palaga

**Affiliations:** 1 Center of Excellence in Immunology and Immune-mediated Diseases, Chulalongkorn University, Bangkok, Thailand; 2 Interdisciplinary Graduate Program in Medical Microbiology, Graduate School, Chulalongkorn University, Bangkok, Thailand; 3 Research Unit of Hepatitis and Liver Cancer, Department of Biochemistry, Faculty of Medicine, Chulalongkorn University, Bangkok, Thailand; 4 Department of Microbiology, Faculty of Medicine, Chulalongkorn University, Bangkok, Thailand; 5 Department of Microbiology, Faculty of Science, Chulalongkorn University, Bangkok, Thailand; Yonsei University, REPUBLIC OF KOREA

## Abstract

Hepatitis virus B (HBV) infection is one of the major causes of hepatocellular carcinomas (HCC). HBx protein encoded in HBV genome is one of the key viral factors leading to malignant transformation of infected cells. HBx functions by interfering with cellular functions, causing aberration in cellular behaviour and transformation. Notch signalling is a well-conserved pathway involved in cellular differentiation, cell survival and cell death operating in various types of cells. Aberration in the Notch signalling pathways is linked to various tumors, including HCC. The role of HBx on the Notch signalling in HCC, however, is still controversial. In this study, we reported that HBV genome-containing HCC cell line HepG2 (HepG2.2.15) expressed higher Notch1 and Delta-like 4 (Dll4), compared to the control HepG2 without HBV genome. This upregulation coincided with increased appearance of the cleavage of Notch1, indicating constitutively activated Notch signalling. Silencing of HBx specifically reduced the level of Dll4 and cleaved Notch1. The increase in Dll4 level was confirmed in clinical specimens of HCC lesion, in comparison with non-tumor lesions. Using specific signalling pathway inhibitors, we found that MEK1/2, PI3K/AKT and NF-κB pathways are critical for HBx-mediated Dll4 upregulation. Silencing of HBx clearly decreased the level of phosphorylation of Akt and Erk1/2. Upon silencing of *Dll4* in HepG2.2.15, decreased cleaved Notch1, increased apoptosis and cell cycle arrest were observed, suggesting a critical role of HBx-Dll4-Notch1 axis in regulating cell survival in HCC. Furthermore, clonogenic assay confirmed the important role of Dll4 in regulating cell survival of HBV-genome containing HCC cell line. Taken together, we reported a link between HBx and the Notch signalling in HCC that affects cell survival of HCC, which can be a potential target for therapy.

## Introduction

Hepatocellular carcinoma (HCC) is considered as the fifth common cancer in the world. Moreover, it is ranked as one of the major causes of cancer-related death because of the rapid progression of tumor and poor prognosis. Various insults to liver can lead to malignant transformation and HCC such as chronic hepatitis B and C virus (HBV/HCV) infection, alcohol abuse, aflatoxin exposure, and non-alcoholic steatohepatitis. Viral HBx is the most critical protein for viral replication in hepatocytes and for development of HCC [1]. HBx is a multifunctional protein that has been shown to regulate many transcription factors such as nuclear factor-kappaB (NF-κB), the activator protein 1 (AP-1), and cAMP response element-binding protein (CREB) and interfere with various signal transduction pathways such as Janus kinase (JAK)/signaling transducer and activator of transcription (STAT), Ras-Raf mitogen-activated protein kinase (MAPK), phosphoinositide-3-kinase-protein kinase B/Akt (PI3K-PKB/Akt), Wnt/β-catenin pathway [2]. These pathways are involved in cellular functions including apoptosis, cell proliferation, cell cycle progression, and cytokine production. Therefore, it is proposed that HBx manipulates these cellular signalling, resulting in transformation of HBV-infected cells. Although extensive studied focused on the roles of HBx in malignant transformation, the molecular mechanisms underlying this process are not well elucidated.

Notch signaling is necessary for various biological processes in multicellular organisms. In mammals, Notch signaling is comprised of the Notch receptors and Notch ligands, i.e. Notch receptors (Notch1-4) and Notch ligands (Jagged1-2, Delta-like (Dll) 1–4). When Notch receptor is engaged by its ligand, this interaction induces a proteolytic cleavage of Notch receptor. The intracellular Notch (ICN) is released from the cell membrane and translocates toward the nucleus for binding to CSL/RBP-Jκ, a DNA binding protein, to form a transcriptional activating complex and regulate transcription of the target genes such as gene in the family of hairy enhancer of split complex (HES) or hairy/enhancer-of-split-related with YRPW motif (HEY) [3]. Aberration in the components of Notch signaling is associated with various types of cancers including HCC [4, 5]. Intriguingly, emerging evidences show that Notch signalling can act as both oncogene and tumor suppressor gene, depending on cellular and signalling context [6]. Therefore, interpretation of data on the roles of Notch in cancer needs to take into consideration of the experimental systems used.

Increase in the level of one of the Notch ligand, Jagged1, was reported in HCC compared with the non-tumor liver tissue and its expression was correlated with that of HBx. This study further showed that both proteins colocalized in HBx overexpression HCC cell line [7]. Another study using human non-tumor hepatic cell line L02 cells to overexpress HBx and found that the components of the Notch signalling is highly upregulated. Treating HBx-overexpressing cells by Notch signalling inhibitor attenuated cell growth, altered cell cycle profiles and induced apoptosis [8]. In the following up study, the same group showed that when overexpressing HBx in HCC cell line HepG2, HBx upregulated mRNA expression of Notch-1, Jagged-1 and Hes-1 by binding to the Notch-1 intracellular domain [9]. Using specific inhibitor to suppress Notch receptor cleavage, it was show that some of this phenotypic changes were partially reversed. In separate study, using HBx overexpressing non-tumor hepatic cell line L02 cell line, Notch1 was demonstrated to co-immunopricipitate with HBx and increasing activation of NF-κB activity in this cell line was shown to be dependent on HBx/Notch [10]. From these studies, the link between HBx and Notch signalling is apparent but the detail molecular mechanisms remained unresolved due to difference in cell line used and most overexpression strategy employed in some studies.

Dll4 is one of the Notch ligands which play a role in angiogenesis in both physiological and pathological states [11]. In cancer, Dll4 is most studied in relationship with tumor angiogenesis [12]. In fact, Dll4 is one of the therapeutic target for cancer treatment [13, 14]. The evidence suggesting the roles of Dll4/Notch in HCC is, however, lacking. In this study, we uncovered the novel link between HBx/Dll4/Notch1 in HCC and elucidated the molecular mechanism resulting in Dll4/Notch1 activation by HBx.

## Materials and Methods

### Cell lines and Reagents

Human hepatocellular carcinoma (HepG2) and HBV genome-transfected HepG2 (HepG2.2.15) cell lines were generous gifts from Professor Antonio Bertoletti (Singapore Institute for Clinical Sciences at Agency for Science, Technology and Research (A*Star)) [15]. Human hepatoma cell line Huh-7 was a gift from Dr. Sanchai Payungporn, Faculty of Medicine Chulalongkorn University (Japanese Collection of Research Bioresources (JCRB), Tokyo, Japan). Human hepatic cell line, THLE-2 (ATCC CRL-2706) were purchased from American Type Culture Collection (Manassas, VA, USA). Cells were maintained according to the providing sources. BAY 11–7082, LY 294002, SB 203580 and U0126 (Billerica, MA, USA) were prepared in dimethyl sulfoxide (DMSO) as stock solutions.

### Human HCC samples

Human liver tissues were obtained from King Chulalongkorn Memorial Hospital. Total RNA were extracted from the samples using RNeasy mini kit (Qiagen, Germany) according to the manufacturer’s instruction.

### Ethics Statement

The use of tissue biopsy was approved by the Institutional Review Board (IRB No. 396/55), Faculty of Medicine, Chulalongkorn University. The written informed consent from the donors was obtained for the use of the samples in this research.

### Quantitative Real-time PCR

Total RNA were extracted using RNeasy mini kit (Qiagen, Germany. The amount of RNA was quantitated by Nanodrop spectrophotometer (Thermo Scientific, USA). Total RNA were converted to cDNA using TaqMan® reverse transcription reagents (Applied Biosystems, USA) in the Thermal Cycler (Eppendorf, USA). Quantitative real-time PCR (qPCR) was carried out using *Power* SYBR® Green PCR Master Mix (Applied Biosystems, USA) and StepOnePlus^TM^ Real-Time PCR Systems (Applied Biosystems, USA). All the primers used in this study are provided as a S1 Table. Relative expression was normalized to the expression of housekeeping gene *actin* by the 2^-ΔΔCT^ method.

### Gene Silencing

The siRNA sequences specific for HBx and Dll4 and scrambled siRNA were designed and purchased from Silencer® Select Pre-Designed and Validated siRNA (Life Technologies, USA). Stealth si-HBx RNA sequences have three duplexes as follows: siHBx-199 5’-AGG TGA AGC GAA GTG CAC ATT-3’ [16], siHBx-260 5’-GAA TGT TGC CCA AGG TCT TAC ATA A-3’ and siHBx-371 5’-GGG AGG AGA TTA GAT TAA AGG TCTT-3’. Stealth si-Dll4 RNA sequences have three duplexes as follows: HSS182569 5’-CCT CTC CAA CTG CCC TTC AAT TTCA-3’, HSS123069 5’-GCC TAT CTG TCT TTC GGG CTG TCAT-3’ and HSS123063 5’-ACC TCC ATT TGT GAT TAG ACA TGTT-3’. Transfection of siRNA was carried out using Lipofectamine RNAiMax Reagent (Life Technologies, USA). After 24–72 hr of transfection, cells were collected for further analysis.

### HBx Overexpression

Cells were transfected with pGFP-HBx (a gift from Xin Wang (Addgene plasmid # 24931)) [17] or control empty vector using X-tremeGENE HP DNA Transfection reagent ((Roche Diagnostics, Germany) according to the instruction by manufacturer. Successful transfection was confirmed by observation under fluorescent microscope and Western blot at 24 hr after transfection. For mRNA analysis by qPCR, cells were sorted for GFP^+^ cells using using FACSAria II (BD Biosciences).

### Western Blot and Flow Cytometry

Cells were treated as indicated and the cell lysates were prepared using lysis buffer (1mM EGTA, 1 mM DTT, 50 mM Tris-HCl pH 7.2, 0.14 M KCl, 2.5 mM MgCl_2_, 0.1% NP-40, phosphatase inhibitor (Sigma-Aldrich, USA) and protease inhibitor (Roche Diagnostics, Germany)). Proteins were measured using Pierce^TM^ BCA Protein Assay Kit (Thermo scientific, USA). The antibodies (Ab) used are as follows: rabbit anti-cleaved Notch1 (Val1744) Ab, rabbit anti-human Notch2 Ab, rabbit anti-human Notch3 Ab and rabbit anti-human Dll4 Ab (Cell Signaling Technology, USA), rabbit anti-Notch1 Ab (C-20) (Santa Cruz Biotechnology, USA), rabbit anti-Notch4 Ab, rabbit anti-human Jagged1 Ab and rabbit anti-human Dll1 Ab (Abcam, USA), mouse anti-HBx Ab (EMD Millipore, USA), mouse anti-actin Ab and rabbit anti-human Hes1 Ab (EMD Millipore, USA), rabbit anti-Erk1/2 Aby, rabbit anti-NF-κBp65 Ab, rabbit anti-Akt antibody and rabbit anti-MAPK p38 Ab (Cell Signaling Technology, USA). The secondary Abs used are as follows: goat anti-rabbit IgG Ab (EMD Millipore, USA) and sheep anti-mouse IgG Ab (Amersham Biosciences, UK). Detection of signals was done using Clarity^TM^ Western ECL Substrate (Bio-Rad, USA) and Hyperfilm^TM^ ECL (Amersham Biosciences, UK). To detect cleaved Notch1 using flow cytometry, cells were fixed in 1.5% formaldehyde and permeablized with cold methanol. Antibodies used to detect cleaved Notch1 was mouse anti-Notch1 (N1A) conjugated with phycoerythrin (Biolegend, USA). Data were acquired on BD FACSCalibur™ and the results were analysed by FlowJo 7.0 software (FlowJo LLC., USA).

### MTS Assay

Cell viability was assayed using CellTiter 96® AQ_ueous_ One solution cell proliferation assay (Promega, USA) following the manufacturer’s instructions.

### Apoptosis and Cell Cycle Analysis

Cells were treated as indicated. Apoptotic cell death was determined using FITC Annexin V Apoptosis Detection kit with PI (Biolegend, USA). For cell cycle analysis, cells were fixed in 70% methanol and treated with RNaseA solution before staining with propidium iodide. Data were acquired using Cytomics FC 500 MPL Flow cytometer system (Beckman Coulter, USA) and analyzed with FlowJo 7.0 software (FlowJo LLC., USA).

### Detection of Viral Replication and Viral Protein Expression

HepG2.2.15 were treated as indicated. DNA was extracted from cell pellets using QIAamp DNA Blood Mini Kit (Qiagen) according to the manufacturer’s instructions. HBV replication was measured by qPCR using StepOnePlus^TM^ Real-Time PCR Systems (Applied Biosystems, USA) compared with the standard plasmid [18]. For normalization to cell numbers, the copy numbers of human *β-globin* gene was used. For qualitative measurement of HBsAg and HBeAg, cells were collected, washed with PBS and lysed in ice-cold lysis buffer after 48 hours of silencing *HBx*. Lysates were cleared by centrifugation. The amount of protein lysates were measured using Pierce^TM^ BCA Protein Assay Kit (Thermo Scientific, USA). The equal amount of protein in each sample was subjected to the ARCHITECT HBsAg Qualitative II or HBeAg 6C32 (Abbott laboratories Ltd, USA) according to the manufacturer’s instructions and the data was analyzed by using the ARCHITECT iSystem.

### Clonogenic Assay

For clonogenic assay, after 24 hour of silencing *Dll4*, 1.5x10^3^ cells were plated in 96 well plates and incubated at 37°C for 5–7 days. At day 7 after the colonies were formed, each well was washed in PBS and fixed with absolute methanol at -20°C for 15 min. Each well was stained with 0.25% crystal violet at room temperature for 30 min. Cells were washed twice by water and dried at room temperature overnight. The number of colonies were observed and counted by light microscope.

### Statistical analysis

Statistical analysis was performed using GraphPad Prism version 5.0 with unpaired student’s t-test. For analysis of gene expression level in HCC biopsy, SPSS 15.0 for Windows with Wilcoxon test was used. Statistical significance was considered when *p* ≤0.05.

## Results

### Expression of the components of the Notch signalling pathways is affected in HBV genome containing human HCC cell line

**Fig 1:**
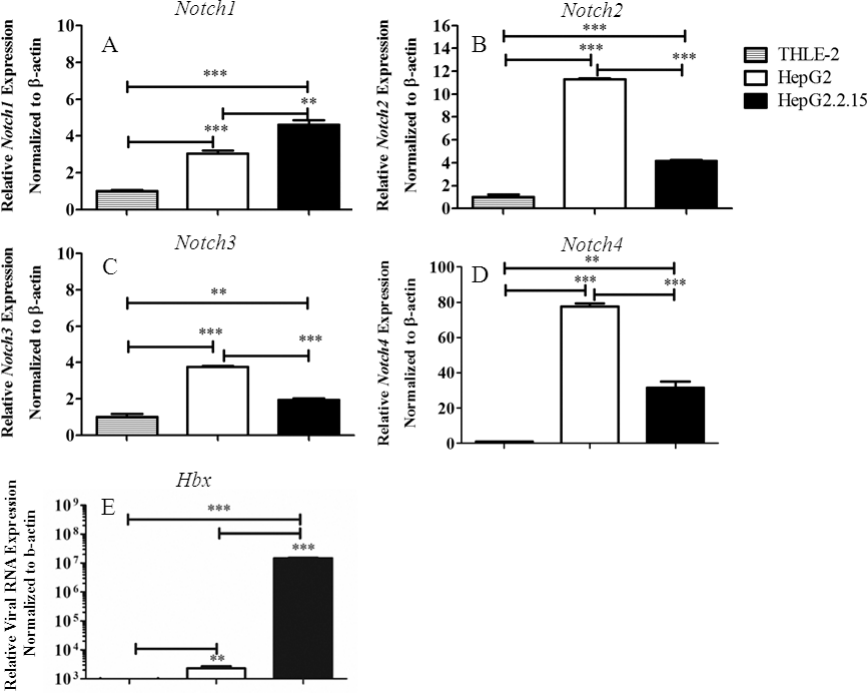
Expression profiles of mRNA of Notch receptors and HBx in THLE-2, HepG2 and HepG2.2.15. Total RNA from three cell lines were analyzed for expression profiles of *Notch1-4* (A-D) and relative viral RNA expression (E) by qPCR. The results are representative of at least two independent experiments where ** and *** indicates statistical significance at the *p* <0.01 and *p*<0.001 level, respectively.

**Fig 2:**
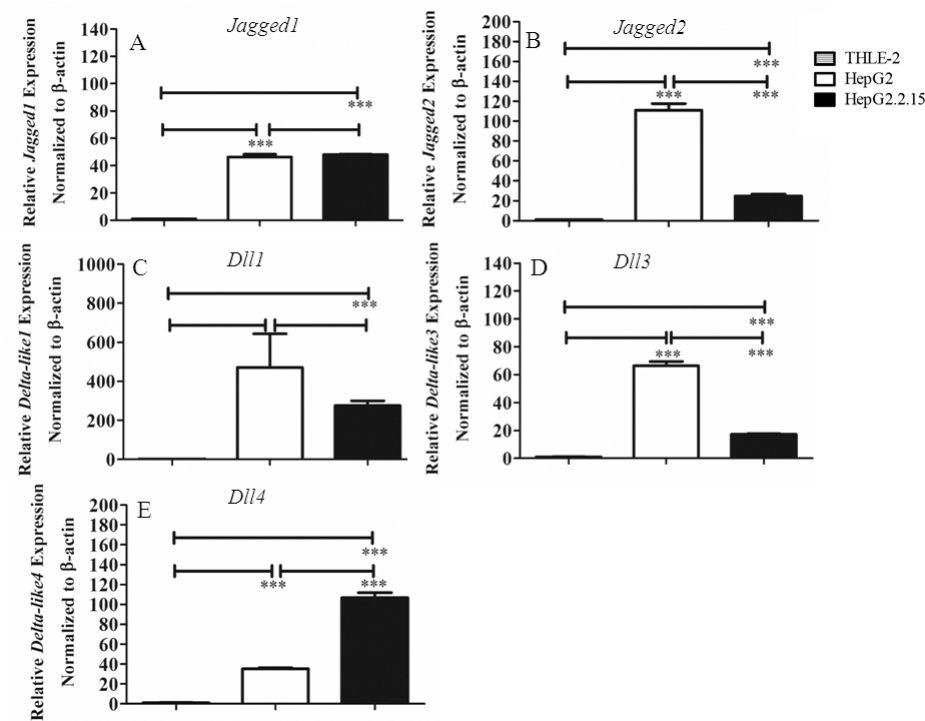
Expression profiles of mRNA of Notch ligands in THLE-2, HepG2 and HepG2.2.15. Total RNA from three cell lines were analyzed for expression profiles of *Jagged 1* and *Jagged2* (A, B) and *Dll1*, *Dll3*, *Dll4* (C-E) by qPCR. The results are representative of at least two independent experiments where *** indicates statistical significance at the *p* <0.001 level.

To address the impact of harboring the HBV genome on the status of Notch signalling, the mRNA level of Notch receptors and Notch ligands were compared in immortalized human hepatocytes THLE-2, human HCC cell line HepG2 and HBV genome transfected HepG2.2.15. As shown in Fig 1, Notch1 is upregulated in HepG2.2.15 compared to the other two cell lines, whereas the expression of other Notch receptors were down-regulated upon HBV genome transfection. For the ligands, Dll4 is significantly upregulated in HepG2.2.15 while the level of other ligands were either unaltered (*Jagged1* and *Dll1*) or down-regulated (*Jagged2* and *Dll3*) in HepG2.2.15 (Fig 2).

**Fig 3:**
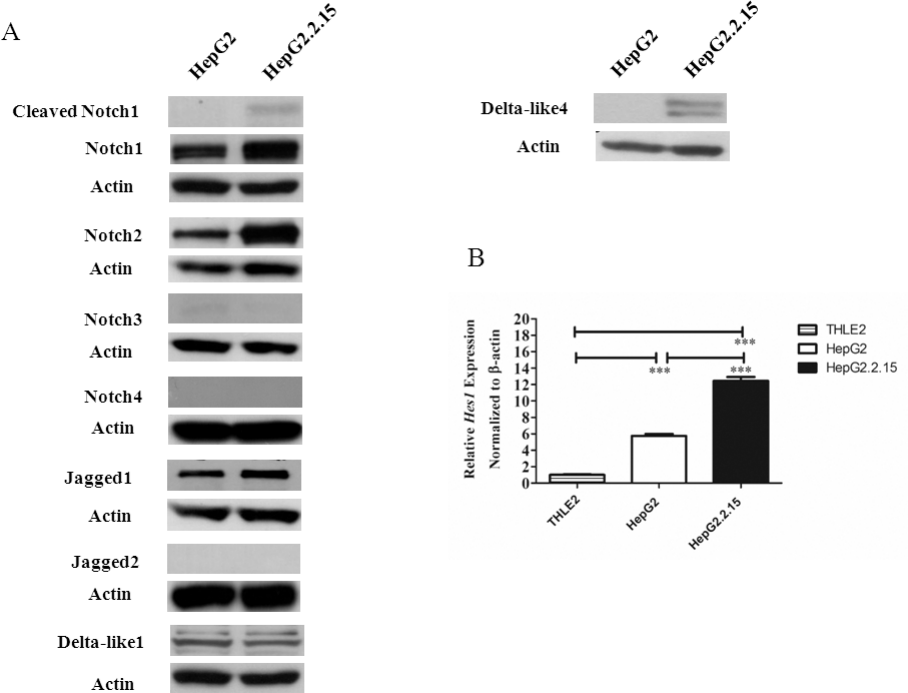
Protein profiles of Notch receptors, Notch ligands and the expression of Notch target gene *Hes1* in HepG2 and HepG2.2.15. (A) Cell lysates from HepG2 or HepG2.2.15 were analysed by Western blot. β-actin was used as loading control. (B) Total RNA from three cell lines were analyzed for expression profiles of *Hes1* by qPCR. The results are representative of at least two independent experiments where *** indicates statistical significance at the *p* <0.001 level.

In order to confirm this observation, Western blots were performed to detect the level of Notch receptors and ligands in HepG2 and Hep2.2.15. As shown in Fig 3A, the cleaved Notch1 (Val1744) was clearly visible in Hep2.2.15. In addition, the protein levels of Notch1, 2 and Dll4 all increased in HBV genome-transfected cell line. Other Notch receptors and ligands were either undetectable or there is no difference between HepG2 and HepG2.2.15 (Fig 3A). Upon activation of Notch signalling, transcription of various target genes are induced, including members of the Hairy Enhancer of Split (Hes) family. When compared among the three cell lines, HepG2.2.15 expressed the highest Hes-1 (Fig 3B). These results together suggest that Notch signalling is activated upon harboring HBV genome and producing HBV, notably Notch1 and Dll4 are the major receptor/ligand of which the level were affected.

### HBx is responsible for activation of Notch signalling via Notch1/Dll4

**Fig 4:**
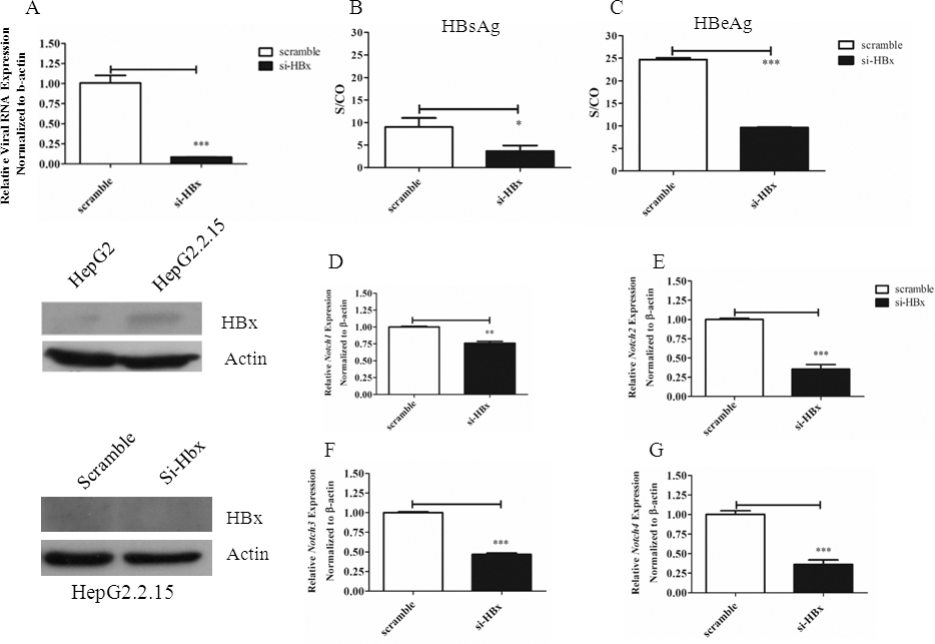
Effects of *HBx* silencing on mRNA and protein expression of HBV viral genes and Notch receptors in HepG2.2.15. HepG2.2.15 was transiently transfected with siRNA specific for *HBx* or control scramble siRNA as described in materials and methods. Total RNA or proteins from HepG2.2.15 transfected with scramble or *HBx* siRNA were analyzed for viral RNA expression or Western blot (A). The relative level of HBsAg (B) and HBeAg (C) was investigated by chemiluminescent microparticle immunoassay (CMIA). S/CO is sample RLU/Cutoff RLU. The level of *Notch 1–4* mRNA was analyzed by qPCR (D-G). The results are representative of at least two independent experiments where *, ** and *** indicates statistical significance at the *p*<0.05, *p* <0.01 and *p*<0.001 level, respectively.

Because HBx plays a critical role in malignant transformation of HBV-infected hepatocytes, we investigated whether HBx is responsible for increasing activation of Notch signalling by siRNA-mediated gene silencing approach. The specific silencing of HBx was demonstrated using qPCR and Western blot (Fig 4A). We also found that this silencing approach affected the expression of other viral proteins, *i*.*e*. HBsAg and HBeAg (Fig 4B and 4C), suggesting that silencing *HBx* also negatively affects other HBV viral protein expression, consistent with previous report [16]. We consistently could only detect the low level of HBx protein by Western blot in HepG2.2.15 (S1 File).

**Fig 5:**
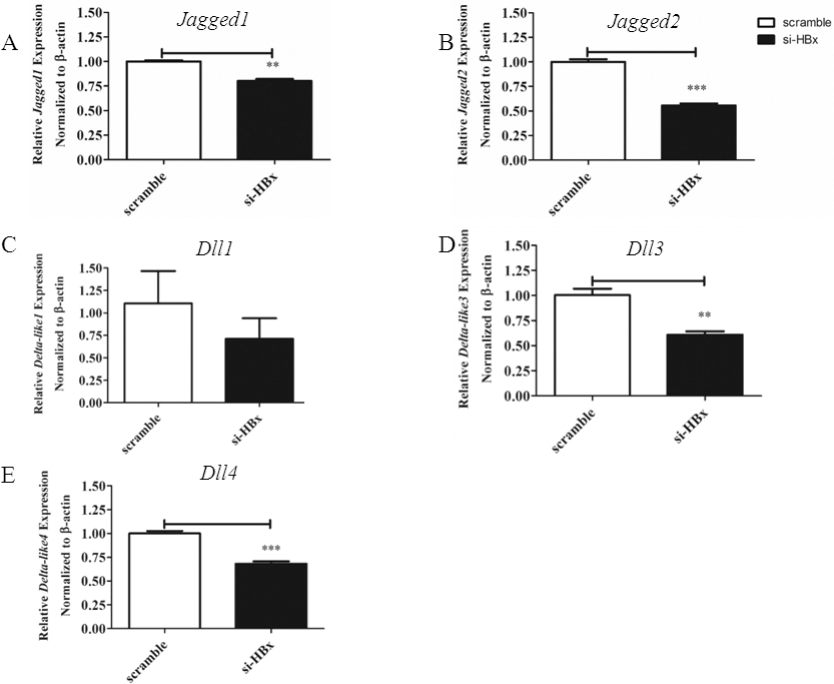
Effects of *HBx* silencing on mRNA expression of Notch ligands in HepG2.2.15. HepG2.2.15 was transiently transfected with siRNA specific for *HBx* or control scramble siRNA as described in materials and methods. Total RNA from three cell lines were analyzed for mRNA expression of *Jagged1* (A), *Jagged 2* (B) *Dll1* (C) *Dll3* (D), *Dll4* (E) by qPCR. The results are representative of at least two independent experiments where ** and *** indicates statistical significance at the *p* <0.01 and *p*<0.001 level, respectively.

**Fig 6:**
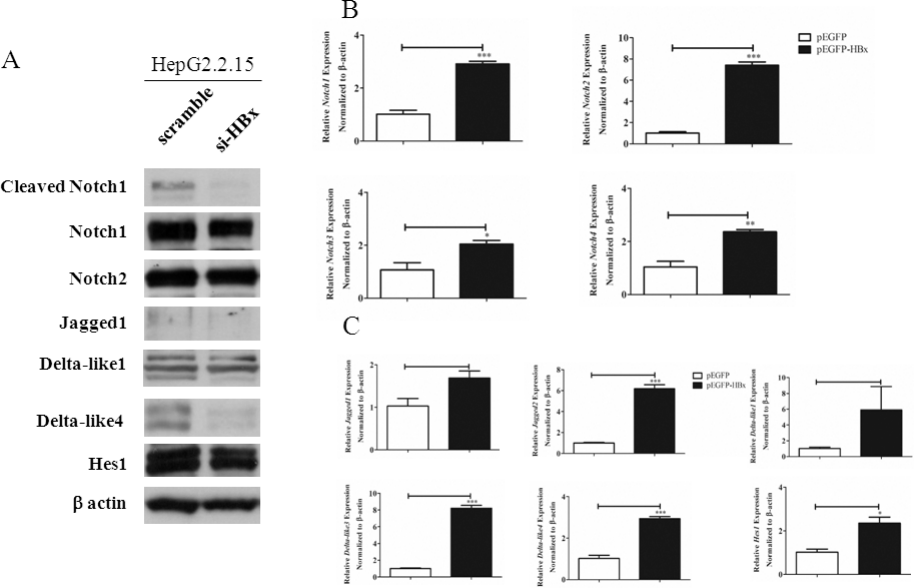
Effects of *HBx* silencing in HepG2.2.15 and HBx overexpression in HepG2 on expression of Notch receptors and ligands. (A) HepG2.2.15 was transiently transfected with siRNA specific for *HBx* or control scramble siRNA as described in materials and methods. Cell lysates from were analysed by Western blot. β-actin was used as loading control. (B-C) HepG2 was transiently transfected with the control pEGFP vector or pEGFP-HBx for 24 hr. Cells were sorted for GFP^+^ cells. Total RNA from three cell lines were analyzed for mRNA expression of Notch receptors, Notch ligands and *Hes1* by qPCR. The results are representative of at least two independent experiments where *, ** and *** indicates statistical significance at the *p* <0.05, *p*<0.01 and *p*<0.001 level, respectively.

As shown in Fig 4D–4G and Fig 5A–5D, silencing of *HBx* globally altered expression level of Notch receptors by downregulating all the receptors. Silencing HBx also reduced the expression of all Notch ligands, except *Dll1* (Fig 5). When Notch receptors and ligands were detected by Western blots in HepG2.2.15 lacking HBx, cleaved Notch1 and Dll4 were disappeared, compared to the control (Fig 6A). In contrast, when HepG2 was transfected to overexpress HBx, all Notch receptors and ligands were upregulated (Fig 6B and 6C). Interestingly, the level of *Hes1*, one of the target genes of Notch signalling, was not affected by HBx silencing in HepG2.2.15 but its mRNA increased in HBx overexpressing HepG2 (Fig 6A and 6B). To investigate whether overexpression of HBx alone is sufficient to trigger activation of Notch, we performed transient transfection of HepG2 by pEGFP-HBx and determined the activation of Notch1 using flow cytometry in GFP^+^ population. HepG2 overexpressing HBx indeed exhibited increased cleaved Notch1 (S2 File). Similar experiment was performed in other human HCC cell line Huh-7 but there was no detectable effect of HBx overexpression in this setting (S2 File). These results are consistent with the reported roles of HBx as a viral factor which interferes with normal cellular processes including transcription and signal transduction. Notch signalling is possibly one of the many cellular proteins that are affected by HBx.

### Dll4 is responsible for activation of Notch signalling in HepG2.2.15

**Fig 7:**
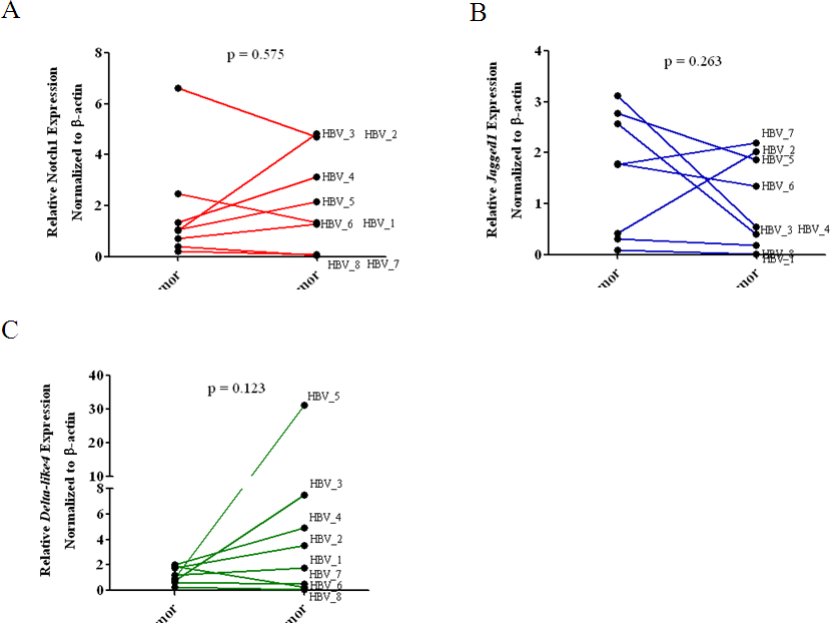
Expression of *Notch1*, *Jagged1* and *Dll4* mRNA in biopsy from HBV-infected HCC patients. Total RNA isolated from dissected biopsy (n = 8) from tumor or non-tumor lesions were analysed for mRNA expression of *Notch1* (A), *Jagged1* (B) and *Dll4* (C) by qPCR.

To confirm our observation in HCC cell lines, we investigated the expression level of *Notch1*, *Jagged1* and *Dll4* in HBV-infected HCC specimens, comparing between tumor and non-tumor lesions (S2 Table). As shown in Fig 7, the mRNA levels of *Notch1* and *Jagged1* were either unchanged or even decreased in tumor lesions compared to the non-tumor lesions (Fig 7A and 7B). Interestingly, 5 out of 8 patients showed a trend of increasing in *Dll4* expression but the difference did not reach statistical significance (Fig 7C). Other Notch receptors and ligands were not analysed in this study because we did not detect any differences between HepG2 and HepG2.2.15 (Fig 3A).

**Fig 8:**
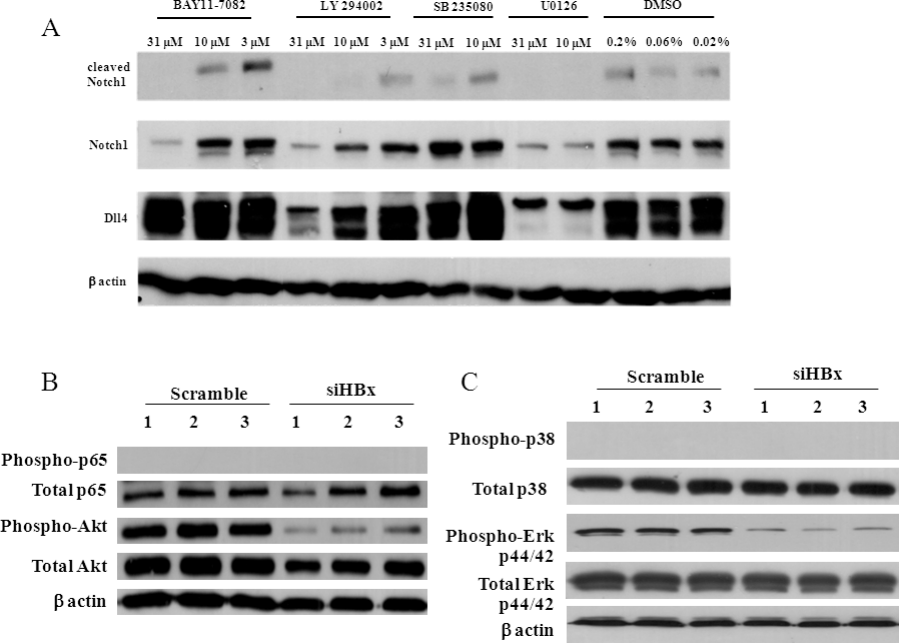
Effects of specific inhibitors on expression of Notch1 and Dll4 and effects of HBx silencing on activation of MAPK, NF-κB and PI3K/Akt pathways in HepG2.2.15. (A) HepG2.2.15 were treated with indicated doses of inhibitors for 24 hr. Cell lysates from were analysed by Western blot. β-actin was used as loading control. BAY11-7082, LY294002, SB203580 and U0126 are pathway specific inhibitors of NF-κB, PI3K/Akt, MAPK/p38 and MAPK/ERK, respectively. (B-C) HepG2.2.15 was transiently transfected with siHBx or control scramble siRNA for 48 hr. Cell lysates from were analysed by Western blot. β-actin was used as loading control.

To gain an insight into the molecular mechanism leading to Dll4 upregulation and activation of Notch1, we treated HepG2.2.15 with various specific signalling pathway inhibitors and measured the level of Notch1, Dll4 and cleaved Notch1. As shown in Fig 8A, inhibition of NF-κB, PI3K/AKT and MEK1/2 showed clear reduction of Notch1. In contrast, only PI3K/AKT and MEK1/2 inhibition showed a significant reduction in Dll4 protein. Consistent with reduction in Notch1 and/or Dll4, the reduction or disappearance of cleaved Notch1 was detected when NF-κB, PI3K/AKT and MEK1/2 were inhibited. To address whether HBx-mediated Dll4 upregulation was via these major pathways, phosphorylation of NF-κB p65, PI3K/AKT, p38 and Erk were investigated when HBx was silenced. As shown in Fig 8B and 8C, HBx silencing significantly decreased the phosphorylation of Erk (p44/42) and Akt while phosphorylated p38 and NF-κB p65 were not detectable in any conditions tested (Fig 8B and 8C). These results suggested that these signalling pathways corroborate in transcriptional activation of Notch1 and Dll4, leading to Notch1 cleavage. Each inhibitor showed differential effects on cell viability of HepG2 and HepG2.2.15 (S1 and S2 Figs).

**Fig 9:**
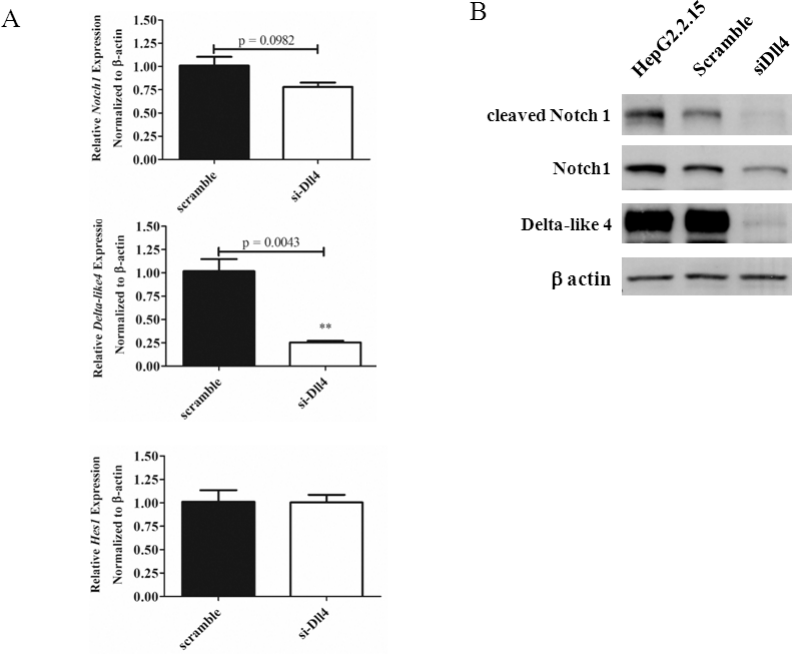
Effects of silencing *Dll4* on the level of cleaved Notch1 in HepG2.2.15. HepG2.2.15 was transiently transfected with siRNA specific for *Dll4* or control scramble siRNA as described in materials and methods. Total RNA was analysed for mRNA expression of *Notch1*, *Dll4* and *Hes1* (A) by qPCR. The results are representative of at least two independent experiments where ** indicates statistical significance at the *p*<0.01 level. Cell lysates were analysed for cleaved Notch1, Notch1 and Dll4 by Western blot. β-actin was used as loading control (B).

To address specifically the role of Dll4, *Dll4* expression was silenced in HepG2.2.15 and investigated its effect. As shown in Fig 9A, silencing *Dll4* slightly reduced the expression of *Notch1* but Notch1 protein was clearly reduced. Furthermore, complete abrogation of cleaved Notch1 was detected upon *Dll4* silencing (Fig 9B). These data strongly indicated that Dll4 is the Notch ligand responsible for Notch activation in HepG2.2.15. We could not, however, rule out the possibility that other Notch ligands may be also involved in activation of Notch1 in HepG2.2.15. In fact, when the mRNA level of one of the target genes of Notch signalling, *Hes1*, was measured by qPCR, no difference was found between control and *Dll4* silencing condition (Fig 9A).

**Fig 10:**
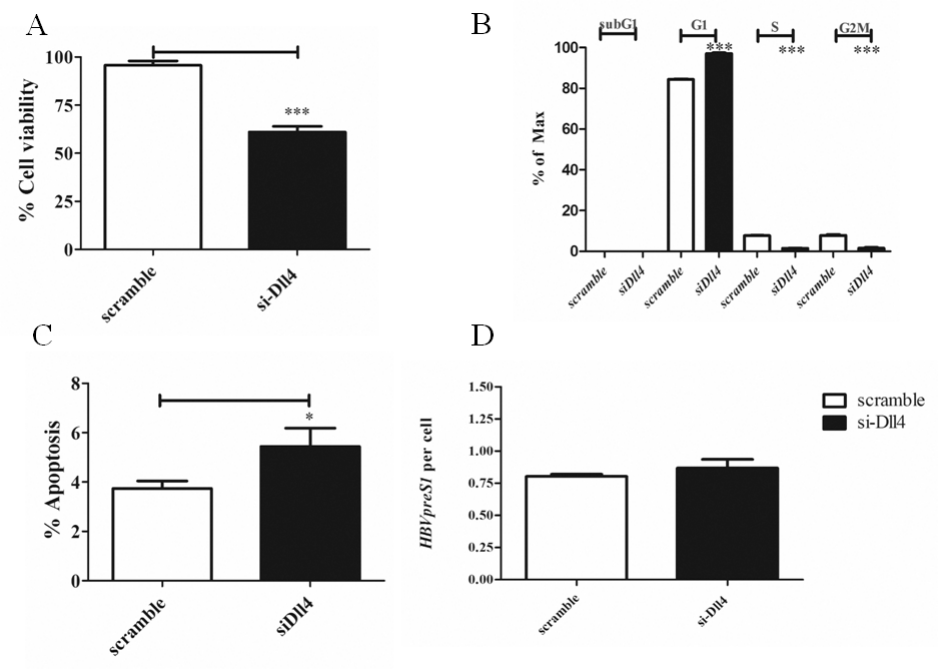
Effect of silencing *Dll4* on cell cycle progression and apoptosis of HepG2.2.15. HepG2.2.15 was transiently transfected with siRNA specific for *Dll4* or control scramble siRNA as described in materials and methods. (A) Cell viability was measured by MTS assay. The results are representative of at least two independent experiments where * and *** indicates statistical significance at the *p* <0.05 and *p*<0.001 level, respectively. (B) Cell cycle analysis was carried out and the percentages of cells in each phase was calculated. The results are representative of at least two independent experiments where *** indicates statistical significance at the *p* <0.001 level. (C) Apoptosis was detected by Annexin V binding assay. The results are representative of at least two independent experiments where * indicates statistical significance at the *p* <0.05 level. (D) HBV genome per cell was measured in control or *Dll4* silencing HepG2.2.15 as described in materials and methods.

The effect of silencing Dll4 was further investigated in cell cycle progression, apoptosis and viral replication. As shown in Fig 10A, silencing of Dll4 in HepG2.2.15 significantly reduced cell viability. Furthermore, Dll4 knockdown led to cell cycle arrest in G1 phase and reduction in percentage of cells in the S and G2/M phase (Fig 10B). Apoptosis was also increased upon *Dll4* silencing (Fig 10C). However, there was no effect on viral replication at the single cell level, suggesting that Dll4/Notch1 interaction affects host cell survival and proliferation without interfering with viral replication (Fig 10D).

**Fig 11:**
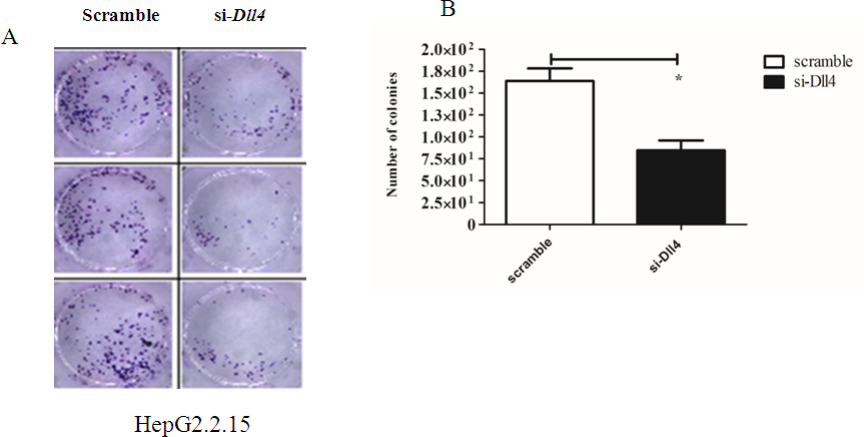
Effect of Silencing Dll4 in HepG2.2.15 in Clonogenic Assay. (A-B) HepG2.2.15 was transfected with siDll4 or scramble siRNA for 24 hr. Cells were subjected to clonogenic assay and the numbers of colonies were measured. * indicates statistical significance at the *p* <0.05 level.

To further characterize the effect of silencing *Dll4* on behaviour of HepG2.2.15 cell line, clonogenic assay was performed. As shown in Fig 11, silencing of *Dll4* in HepG2.2.15 significantly reduced the numbers of colony detected in the clonogenic assay (Fig 11A and 11B. These results strongly indicated that Dll4 plays important role in survival and malignant transformation of HepG2.2.15 cell line.

## Discussion

Regulatory protein HBx interferes with various signalling pathways and cellular target proteins that results in transformation of infected cells [2, 19]. Consistent with previous reports, we found that Notch signaling is under the regulation of HBx [8, 9, 20, 21]. Despite reports on the crosstalk between HBx and Notch signalling, there are some contradictory and inconsistent results. Therefore, to address how HBV infection leads to activation of Notch signaling, we investigated the complete profiles of Notch receptors and ligands expression in HCC cell line HepG2.2.15, a cell line derived from transfection of HepG2 with whole HBV genome. In this cell line, increased *Notch1*, *Dll4* and *Hes1* expression were observed, compared to the parental cell line HepG2 and immortalized hepatic cell line THLE-2. Interestingly, HepG2 showed the highest level of *Notch2* mRNA while HepG.2.215 has the highest Notch2 protein (Figs 1B and 3A). This discrepancy may be due to high *Notch2* mRNA turnover rate in HepG2 or the difference in the stability of protein Notch2 between the two cell lines. In fact, Notch receptors in the full length form or membrane-tethered form can be targeted for degradation by proteasome, a process that is proposed to be mediated by Numb [22]. More importantly, cleaved Notch1 was found only in HepG2.2.15, indicating that Notch signaling is activated. Using gene silencing approach in HepG2.2.15, Dll4 and Notch1 were found to be selectively induced by HBx.

Previous studies from Wang *et al*. found that cleaved Notch1, Notch1 and Hes1 were up-regulated in *HBx*-transfected HepG2 cell lines compared with HepG2 and this data showed that the activation of Notch1 was regulated by HBx [9]. This report is consistent with our obtained data. However, they further showed that Jagged1 was increased in *HBx*-transfected HepG2 but there was no data on the level of Dll4. Furthermore, Gao *et al*. noted that *Jagged1* expression was upregulated in *HBx*-transfected HepG2 cells whereas it was downregulated when silencing *HBx* in HepG2.2.15 [7]. These results are in contrast with our data that demonstrated that Dll4, but not Jagged1, is specifically downregulated when HBx is silenced in HepG2.2.15. The involvement of HBx in Dll4 upregulation was further confirmed by the siRNA silencing of HBx in HepG2.2.15. Silencing Dll4 completely abrogated the appearance of cleaved Notch1, strongly indicated that Dll4 is responsible ligand to engage with Notch1 in HepG2.2.15. The discrepancy between our results and previous reports may be due to difference in the experimental design using HBx overexpression in those study while we used HBV genome transfection HCC cell line. In our hand, HBx overexpression in HepG2 globally increased all components of Notch signaling, including Jagged1 and Dll4 and later massive cell death was observed. But because expression of Jagged1 and Dll1 were also present in HepG2.2.15, we could not rule out the possibility that these two ligands are involved in activating Notch receptors.

Expression of HBx was only detectable in HepG2.2.15 at the mRNA level by semi-quantitative RT-PCR but not Western blot in this study. When cells were transiently transfected to overexpress HBx, the protein could be detected but cell death were later observed. These results suggested that the expression level of HBx is tightly controlled and high level of HBx in HepG2 cell line may be toxic to cells. It was reported that HBx is expressed at low level during acute and chronic hepatitis [23]. Using siRNA approach to knockdown HBx as described by Han *et al*., we observed a decrease in mRNA level of *Pol* of HBV [16]. This is consistent with the previous report that silencing *HBx* also affects HBV gene expression and reduces viral replication.

The transient overexpression of HBx alone at early time point (24 hr) in HepG2 cell line increased the level of cleaved Notch1 (S2 File), indicating that HBx alone is sufficient in inducing Notch activation. However, the similar approach in another human HCC cell line Huh-7 did not show any increase in cleaved Notch1, Notch1 or Dll4 (S2 File). This discrepancy may indicate an underlying differences between the two cell lines.

One of the Notch target genes, *Hes1*, is increased in HBV genome containing HepG2.2.15, compared to the parental cell line HepG2 (Fig 3B) and its level was increased when HBx was overexpressed in HepG2 (Fig 6C). In contrast, the level of *Hes1* in *HBx*-knockdown and *Dll4*-knockdown HepG2.2.15 did not change (Figs 6A and 9A). These results indicated that the level of *Hes1* may be under the control of other Notch receptor/ligand interaction via other HBV genes in HepG2.2.15.

To confirm our observation in HBV genome transfected HCC cell line, we studied the expression of *Notch1*, *Jagged1*, and *Dll4* in HBV-infected HCC tissue biopsy by qPCR. Our result showed that tumor lesions in HBV-infected HCC have increased level of *Notch1* (50%), *Dll4* (62.5%), and *Jagged1* (12.5%), compared to non-tumor lesions. Although the numbers of specimens studied were small and the difference in *Dll4* level between tumor lesions vs. non-tumor lesions did not reach statistical significance, this result was in contrast with the report from the previous study. This discrepancy may be due to the difference in stage of tumor, genotype of HBV, treatments patients received before liver biopsy.

From our result, it indicated that the activation of Notch signalling was regulated by HBx via Notch1/Dll4 axis. We further detected the effect of Notch inhibition on cell viability, apoptosis, cell cycle and viral load upon silencing Dll4 in HepG2.2.15. The results revealed that HepG2.2.15 decreased cell viability and increased apoptosis similar to the result obtained by gamma secretase inhibitor (GSI) treatment reported by Wang et al. [8]. Interestingly, Notch inhibition by silencing *Dll4* in HepG2.2.15 led to cell cycle arrest at G1 phase in our study whereas GSI treatment of human non-tumor hepatic cell line L02 cells resulted in shorten S phase and increased apoptosis [8]. In addition, we detected the effect of inhibition of Notch signalling on viral replication and found that the activation of Notch signalling did not regulate viral replication. Because silencing *HBx* decreased HBV viral replication but silencing *Dll4* did not show defect in viral replication, HBx may regulate viral replication independently of Dll4.

Using multiple specific pathway inhibitors, we investigated which downstream pathway(s) of HBx is responsible for the activation of Notch signalling. The results revealed that Erk (MEK1/2) and PI3K/Akt pathways were the major pathways to control Notch activation downstream of HBx via induction of Dll4 expression. Furthermore, NF-κB, Erk (MEK1/2) and PI3K/Akt were all critical for Notch1 expression. HBx has been linked to act upstream of Erk and NF-κB pathways and Notch signalling is shown to crosstalk with NF-κB pathways in HBx-overexpressing L02 cells [2, 10]. We presented evidence here that HBx also control PI3K/Akt and regulates Notch1 expression. The phosphorylation of NF-κB p65 subunit was not detectable in HepG2.2.15 cell line but the NF-κB specific inhibitor suppressed HBx-mediated Dll4 upregulation. Therefore, other NF-κB subunit(s) may be involved in this regulation such as NF-κBp52 [24].

Many previous studies showed that Dll4 expression were mostly found on the endothelial cells and it was considered as the mediator of Notch activation of angiogenesis in many cancers such as breast, colon, and pancreatic cancers [12, 14]. Targeting Dll4 is considered one of the new anti-cancer therapy [14]. Taken together, our data provides a novel link between HBx and Dll4/Notch1 in HCC via Erk and PI3K/Akt pathways. Therefore, Dll4 could be used as new target for therapy for HBV-infected HCC.

## Supporting Information

S1 Fig Effect of specific pathway inhibitors to cell viability in HepG2 and HepG2.2.15 cell lines at 24 hr after treatment. (DOCX)

S2 Fig Effect of specific pathway inhibitors to cell viability in HepG2 and HepG2.2.15 cell lines at 96 hr after treatment. (DOCX)

S1 File Detection of HBx mRNA transcripts and protein in HepG2.2.15. (DOCX)

S2 File Overexpression of HBx in HepG2 and Huh7. (DOCX)

S1 Table List of primers used in this study. (DOCX)

S2 Table Clinicopathological parameter in HCC patients. (DOCX)
