# Supplementary material for: Hepatitis B Virus HBx Activates Notch Signaling via Delta-Like 4/Notch1 in Hepatocellular Carcinoma
Source: PLoS One. 2016 Jan 14;11(1):e0146696. doi: 10.1371/journal.pone.0146696 (PMC4713073; doi:10.1371/journal.pone.0146696)
Supplement: S1 Fig — (DOCX) [file pone.0146696.s001.docx]

**S1 Fig.** Effect of specific pathway inhibitors to cell viability in HepG2 and HepG2.2.15 cell lines at 24 hr after treatment

Cells were treated with various doses of inhibitors for 24 hr. Cell viability was determined by MTS assay. The results represent mean±SEM and representative of one independent experiments. DMSO was used as vehicle control.
