# Supplementary material for: Hepatitis B Virus HBx Activates Notch Signaling via Delta-Like 4/Notch1 in Hepatocellular Carcinoma
Source: PLoS One. 2016 Jan 14;11(1):e0146696. doi: 10.1371/journal.pone.0146696 (PMC4713073; doi:10.1371/journal.pone.0146696)
Supplement: S1 File — (DOCX) [file pone.0146696.s003.docx]

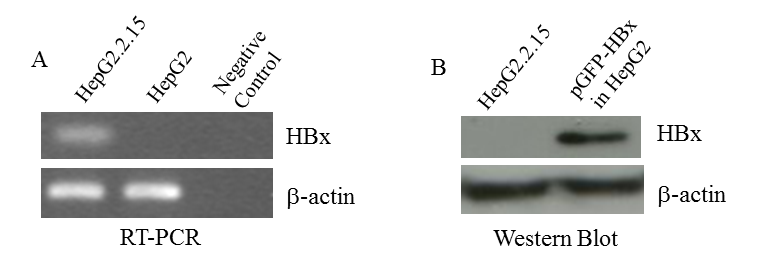


**S1 File.** Detection of *HBx* mRNA transcripts and protein in HepG2.2.15.

(A) Viral RNA expression was detected in HepG2.2.15 or HepG2 cell line by RT-PCR. Negative control represented RT-PCR reaction using mRNA from HepG2.2.15 cell line without addition of reverse transcriptase. (B) HBx protein was detected in HepG2.2.15 cell line or HBx-overexpressing HepG2 cell line by Western blot.
