# Supplementary material for: Hepatitis B Virus HBx Activates Notch Signaling via Delta-Like 4/Notch1 in Hepatocellular Carcinoma
Source: PLoS One. 2016 Jan 14;11(1):e0146696. doi: 10.1371/journal.pone.0146696 (PMC4713073; doi:10.1371/journal.pone.0146696)
Supplement: S2 File — (DOCX) [file pone.0146696.s004.docx]

**
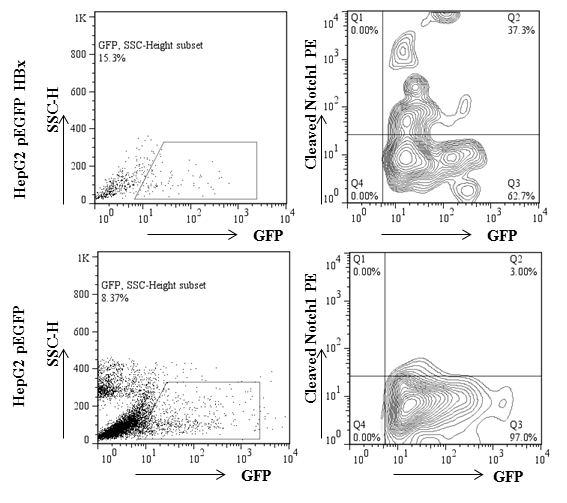

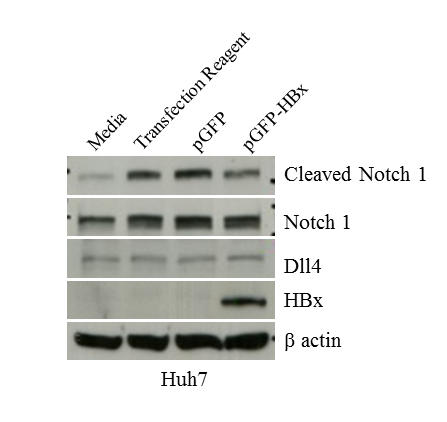
**

A B

**S2 File.** Overexpression of HBx in HepG2 and Huh7

(A) HepG2 was transiently transfected with pEGFP-HBx or control empty vector for 24 hr and the expression of cleaved Notch1 (anti-Notch1 N1A PE Ab) was detected in GFP^+^ population by flow cytometry. Gated cells based on side scatter (SSC) and GFP (left panel) were further analysed for cleaved Notch1 (right panel. (B) Human hepatoma cell line, Huh7, was transiently transfected with pEGFP-HBx or control empty vector for 48 hr. Expression of cleaved Notch1 (Val1744), Notch1, Dll4 and HBx were detected by Western blot.
