# Supplementary material for: Hepatitis B Virus HBx Activates Notch Signaling via Delta-Like 4/Notch1 in Hepatocellular Carcinoma
Source: PLoS One. 2016 Jan 14;11(1):e0146696. doi: 10.1371/journal.pone.0146696 (PMC4713073; doi:10.1371/journal.pone.0146696)
Supplement: S1 Table — (DOCX) [file pone.0146696.s005.docx]

**Supporting Information Captions**

**S1 Table.**

List of primers used in this study

| Gene | Primer Sequence |
| --- | --- |
| *Notch1* | (forward) 5’-CAG CCT GCA CAA CCA GAC AGA-3’  (reverse) 5’-TGA GTT GAT GAG GTC CTC CAG-3’ |
| *Notch2* | (forward) 5’-TGA GTA GGC TCC ATC CAG TC-3’  (reverse) 5’-TGG TGT CAG GTA GGC ATG CT-3’ |
| *Notch3* | (forward) 5’-TCT TGC TGC TGG TCA TTC TC-3’  (reverse) 5’-TGC CTC ATC CTC TTC AGT TG-3’ |
| *Notch4* | (forward) 5’-CAC TGA GCC AAG GCA TAG AC-3’  (reverse) 5’-ATC TCC ACC TCA CAC CAC TG-3’ |
| *Jagged1* | (forward) 5’-AAG GGG TGC GGT ATA TTT CC-3’  (reverse) 5’-TCC CGT GAA GCC TTT GTT AC-3’ |
| *Jagged2* | (forward) 5’-AAT GGT GGC ATC TGT GTT GA-3’  (reverse) 5’-GCG ATA CCC GTT GAT CTC AT-3’ |
| *Dll1* | (forward) 5’-CCA CGC AGA TCA AGA ACA CC-3’  (reverse) 5’-GGA TGA GTG CGT CAT AGC AA-3’ |
| *Dll3* | (forward) 5’-TTC CCT ACC CTT CCT CGA TT-3’  (reverse) 5’-ATG GCA GGT AGC TCA AAA CG-3’ |
| *Dll4* | (forward) 5’-GCG AGA AGA AAG TGG ACA GG-3’  (reverse) 5’-ACA GTA GGT GCC CGT GAA TC-3’ |
| *HBx* | (forward) 5’-CAC CTC TCT TTA CGC GGA CT-3’  (reverse) 5’-GGT CGT TGA CAT TGC AGA GA-3’ |
| *Hes1* | (forward) 5’-ACG ACA CCG GAT AAA CCA AA-3’  (reverse) 5’-CGG AGG TGC TTC ACT GTC AT-3’ |
| *Actin* | (forward) 5’- ACC AAC TGG GAC GAC ATG GAG AA -3’  (reverse) 5’-GTG GTG GTG AAG CTG TAG CC-3’ |
