# Supplementary material for: Hepatitis B Virus HBx Activates Notch Signaling via Delta-Like 4/Notch1 in Hepatocellular Carcinoma
Source: PLoS One. 2016 Jan 14;11(1):e0146696. doi: 10.1371/journal.pone.0146696 (PMC4713073; doi:10.1371/journal.pone.0146696)
Supplement: S2 Table — (DOCX) [file pone.0146696.s006.docx]

**S2 Table.**

Clinicopathological parameter in HCC patients

| ID | Age (years) | Sex | Tumor Size (cm) | Differentiation | TNM stage | HBSAg | HBeAg | Anti-HBe |
| --- | --- | --- | --- | --- | --- | --- | --- | --- |
| 1 | 63 | Male | 26x16x12 | Poorly | IIIA | Positive | Negative | Positive |
| 2 | 46 | Male | 15x17x9 | Poorly | IIIA | Positive | Positive | Negative |
| 3 | 69 | Male | 1.7x1.5x1.2 | Poorly | IIIB | Positive | Negative | Positive |
| 4 | 53 | Male | 2.2x2.2x1.5 | Moderately | IIIB | Positive | Negative | Positive |
| 5 | 52 | Male | 4.5x4x4 | Well | I | Positive | Negative | Positive |
| 6 | 49 | Male | 10x9x5.5 | Poorly | IIIA | Positive | Negative | Positive |
| 7 | 68 | Male | 3.5x3x2.2 | Well | II | Positive | Negative | Positive |
| 8 | 48 | Male | 13x8.5x7 | Moderately | IIIB | Positive | Negative | Positive |
